# Clinical significance of plasma SERPINA3 in patients with missed miscarriage

**DOI:** 10.3389/fmed.2026.1838072

**Published:** 2026-05-29

**Authors:** Yaping Jiang, Anna Song, Tao Liu, Xiaoqian Nie, Hui Wang, Li Zhao, Qiaoqiao Kong, Huanying Lou, Lingmei Wang, Haifeng Wang, Bo Zhang

**Affiliations:** 1 Department of Reproductive Genetics, Taian Central Hospital Affiliated to Qingdao University, Taian, Shandong, China; 2 Gynecology Minimally Invasive Surgery Center, Taian Central Hospital Affiliated to Qingdao University, Taian, Shandong, China; 3 Department of Cardiothoracic Surgery, Taian Central Hospital Affiliated to Qingdao University, Taian, Shandong, China; 4 Department of Psychology, Shandong Provincial Hospital Affiliated to Shandong First Medical University, Jinan, Shandong, China

**Keywords:** biomarkers, missed miscarriage, normal pregnancy, ROC analysis, SERPINA3

## Abstract

**Background:**

Missed miscarriage is a frequent form of early pregnancy loss, yet its pathophysiology remains incompletely understood and sensitive blood-based biomarkers for detection of established missed miscarriage are limited.

**Methods:**

In this single-center case-control study, we enrolled women with ultrasound-confirmed first-trimester missed miscarriage, women with viable intrauterine pregnancies undergoing elective induced abortion, and non-pregnant women of reproductive age. Circulating serine protease inhibitor A3 (SERPINA3) was quantified by ELISA. Routine hematological, biochemical, and coagulation parameters were collected. Associations with SERPINA3 were assessed using Spearman correlation and multivariable regression, and diagnostic performance was evaluated by receiver operating characteristic (ROC) analysis.

**Results:**

Circulating SERPINA3 differed significantly across the three groups (missed miscarriage, *n* = 91; normal pregnancy, *n* = 83; non-pregnant controls, *n* = 66; Kruskal-Wallis *P* < 0.001), with the highest levels in missed miscarriage, intermediate levels in the normal pregnancy group, and the lowest levels in non-pregnant controls. Compared with missed miscarriage, the normal pregnancy group showed higher systolic blood pressure (*P* = 0.022) and AST/ALT ratio (*P* = 0.001), but lower ALT and GGT levels (both *P* = 0.001), and eosinophil percentage and absolute eosinophil count also differed (*P* = 0.003 and *P* = 0.014). After adjustment for age, BMI, systolic blood pressure, ALT, GGT, and eosinophil count, SERPINA3 remained independently associated with pregnancy outcome (adjusted OR = 1.018, 95% CI: 1.010–1.025, *P* < 0.001).Within the missed miscarriage group, higher SERPINA3 (above median) was associated with higher lymphocyte counts (*P* = 0.042) and fibrinogen concentrations (*P* = 0.014) after adjustment for age and BMI. SERPINA3 correlated positively with GGT, white blood cell count, eosinophil indices, and lymphocyte count; GGT and eosinophil count remained independently associated with SERPINA3 in linear regression.SERPINA3 discriminated missed miscarriage from normal pregnancy controls with an AUC of 0.8751 (95% CI: 0.7641 to 0.9153, *P* < 0.0001), yielding 85.54% sensitivity and 72.53% specificity.

**Conclusion:**

SERPINA3 is markedly elevated in missed miscarriage and serves as an indicator of established missed miscarriage, and its predictive value for future loss remains to be confirmed in prospective studies. Prospective multicenter validation and mechanistic studies are warranted.

## 1 Introduction

Missed miscarriage refers to a condition in which the embryo or fetus dies but the pregnancy tissue is not expelled in a timely manner, and it is typically confirmed in early pregnancy by ultrasound examination (1–3). As an important subtype of early pregnancy loss, its incidence is approximately 10%−15% (4–6), and it may lead to complications such as uterine bleeding, infection, intrauterine adhesions, and coagulation abnormalities (6–9). Beyond physical harm, some patients may also experience psychological stress responses such as anxiety and depression after the diagnosis of miscarriage, with sustained negative impacts on quality of life (10–12).

Among the etiologies of missed miscarriage, embryonic chromosomal abnormalities account for approximately 56%−60% (13). The remaining cases may be associated with infection, immune imbalance, thrombophilia, uterine structural abnormalities, or endocrine disorders (3, 14–18). However, a substantial proportion of cases still lack an identifiable cause. Current clinical diagnosis mainly relies on ultrasound and dynamic monitoring of β-hCG; when imaging findings are inconclusive, markers such as progesterone can serve as supportive evidence. Nevertheless, existing serological markers have limited sensitivity and specificity for early, asymptomatic missed miscarriage, and therefore cannot adequately meet the needs of risk stratification and detection of established missed miscarriage. Accordingly, exploring circulating biomarkers that reflect underlying pathological mechanisms is of significant clinical value.

Inflammation and disruption of immune homeostasis are considered to play a pivotal role in pregnancy failure. Maintenance of pregnancy depends on the precise temporal and spatial regulation of pro-inflammatory and anti-inflammatory signals (19). The serine protease inhibitor (serpin) family participates in a wide range of biological processes—including inflammation, coagulation, and tissue remodeling—by regulating proteolytic activity (20–22). SERPINA3 (α1-antichymotrypsin), an acute-phase reactant, inhibits multiple proteases released by neutrophils and mast cells and has anti-inflammatory, antioxidant, and anti-angiogenic functions (23–26). It is markedly elevated under systemic inflammatory conditions (27), yet its expression profile and clinical relevance in missed miscarriage have not been systematically evaluated.

Therefore, this study aims to characterize changes in plasma SERPINA3 levels in women with missed miscarriage during early pregnancy, analyze its associations with inflammation- and metabolism-related indicators, and evaluate its diagnostic performance as a potential serological biomarker.

## 2 Methods

### 2.1 Study design and participants

This single-center case-control study was conducted at Taian Central Hospital (Taian, China) from January 2026 to March 2026. Participants were allocated into three groups: (1) women with ultrasound-confirmed first-trimester missed miscarriage (case group), (2) women with viable intrauterine pregnancies (normal pregnancy group), and (3) non-pregnant women of reproductive age with regular menstrual cycles (non-pregnant control group).

### 2.2 Diagnostic criteria

**Missed miscarriage** was defined as: (i) an empty gestational sac with mean sac diameter ≥25 mm without a yolk sac, or a crown–rump length ≥7 mm without fetal cardiac activity on transvaginal ultrasound; and (ii) absence of clinical miscarriage symptoms (e.g., vaginal bleeding or abdominal pain). Diagnostic criteria were based on published recommendations. (4, 28–30)

**Normal pregnancy** was defined as an intrauterine gestational sac/embryo consistent with gestational age and/or visible fetal cardiac activity on ultrasound.

### 2.3 Eligibility criteria

**Inclusion criteria (case group):** (1) met the diagnostic criteria for missed miscarriage; (2) gestational age ≤ 12 weeks; (3) maternal age ≤ 35 years.

**Inclusion criteria (normal pregnancy group):** (1) met the diagnostic criteria for normal pregnancy; (2) gestational age ≤ 12 weeks; (3) maternal age ≤ 35 years.

**Inclusion criteria (non-pregnant control group):** (1) non-pregnant women with regular menses; (2) age ≤ 35 years.

**Exclusion criteria (all groups):** (1) known parental chromosomal abnormalities documented in medical records; (2) severe cardiac, hepatic, or renal dysfunction; (3) incomplete clinical data; (4) severe psychiatric disorders with poor adherence; (5) history of malignancy; (6) acute inflammatory conditions or active infection at enrollment.

### 2.4 Clinical data collection

Demographic and clinical information was extracted from the electronic medical record system, including age, body mass index (BMI), obstetric history, and routine laboratory parameters (complete blood count, liver and renal function tests, and coagulation indices). Gestational age was calculated from the first day of the last menstrual period and confirmed or corrected by ultrasound. Urea, creatinine, and uric acid were not determined in the normal pregnancy group due to differences in routine laboratory testing at sample collection. These parameters were only available for the missed miscarriage group and were used for subgroup analysis.

### 2.5 Blood sampling and plasma preparation

Fasting peripheral venous blood samples were collected at enrollment before pregnancy termination (where applicable). Samples were centrifuged at 3,000 × g for 15 min. Plasma was separated and stored at −80°C until analysis.

### 2.6 Measurement of plasma SERPINA3 by ELISA

Plasma SERPINA3 concentrations were quantified using a commercial ELISA kit (catalog No. 11056901H4259; Youpin Biotech, China). All samples were measured in duplicate (technical replicates), and the mean value was used for statistical analyses. Assays were performed according to the manufacturer's instructions.

### 2.7 Psychological assessment

Anxiety and depressive symptoms were assessed at enrollment using the self-administered Generalized Anxiety Disorder-7 (GAD-7) and Patient Health Questionnaire-9 (PHQ-9). These measures were included as exploratory variables to characterize potential coexisting psychological symptoms rather than as primary study outcomes.

## 3 Statistics

Statistical analyses were performed using SPSS software. Continuous variables were first assessed for normality using the Shapiro–Wilk test. Normally distributed data are presented as mean ± standard deviation, whereas non-normally distributed data are expressed as median (interquartile range). Comparisons among three groups were conducted using the Kruskal–Wallis test, followed by *post-hoc* pairwise comparisons with Bonferroni correction when appropriate. Comparisons between two groups were performed using the Mann–Whitney *U*-test for non-normally distributed variables.

Correlations between SERPINA3 levels and clinical or laboratory parameters were evaluated using Spearman correlation analysis. Variables showing significant correlations were further examined using linear regression analysis to identify independent associations with SERPINA3 concentration. Binary logistic regression analysis was applied to assess whether selected variables were independently associated with high SERPINA3 levels. Receiver operating characteristic (ROC) curve analysis was performed to evaluate the diagnostic performance of SERPINA3, and the area under the curve (AUC), sensitivity, and specificity were calculated. All statistical tests were two-sided, and a *P* value < 0.05 was considered statistically significant.

## 4 Results

### 4.1 Comparison of baseline characteristics between the missed miscarriage and normal pregnancy groups

The baseline demographic and laboratory characteristics of the missed miscarriage and normal pregnancy groups are summarized in Table 1. No significant differences were observed in age, BMI, heart rate, diastolic blood pressure, or most hematological and coagulation parameters. However, systolic blood pressure and several liver-related indices differed significantly between groups.

**Table 1:** Baseline demographic and laboratory characteristics of participants in the missed miscarriage and normal pregnancy groups.

	Missed miscarriage group (*n* = 91)	Normal pregnancy group (*n* = 83)	*P*
Demographics			
Age (years)	29.0 (27.0, 36.0)	31.5 (26.25, 36.0)	0.058
BMI (kg/m^2^)	22.86 (20.03, 24.84)	22.72 (21.38, 24.22)	0.222
Heart rate, bpm	78 (72, 82)	78 (73, 80)	0.256
Systolic blood pressure (mmHg)	115.78 ± 9.499	119.74 ± 9.781	**0.022**
Diastolic blood pressure, mmHg	74 (69, 79)	75 (70, 78)	0.686
Laboratory tests			
AST/ALT	1.0066 ± 0.45663	1.3231 ± 0.55933	**0.001**
WBC (×10^9^/L)	7.0189 ± 1.4874	7.5004 ± 1.9707	0.071
Neutrophil percentage (%)	66.4 ± 6.8589	66.778 ± 7.0925	0.724
Lymphocyte percentage (%)	25.552 ± 5.6384	25.262 ± 6.3901	0.753
Neutrophil count	4.7033 ± 1.25523	5.0764 ± 1.62084	0.097
Red blood cell count (RBC)	4.2883 ± 0.34298	4.329 ± 0.2876	0.405
Hematocrit (HCT)	0.37534 ± 0.029645	0.37837 ± 0.025671	0.479
Platelet count (PLT)	251.464 ± 59.0186	246.346 ± 48.4526	0.539
Prothrombin time (PT)	12.8 (12.4, 13.1)	12.6 (12.3, 13.0)	0.356
International normalized ratio (INR)	1.07 (1.04, 1.10)	1.05 (1.03, 1.09)	0.349
Prothrombin time ratio (PTR)	1.07 (1.03, 1.09)	1.05 (1.03, 1.08)	0.365
Prothrombin activity (PTA)	93.8 (91.6, 96.8)	95.2 (92.3, 97.57)	0.345
Alanine aminotransferase (ALT, U/L)	12.80 (9.30, 24.00)	11.05 (8.43, 22.28)	**0.001**
Aspartate aminotransferase (AST, U/L)	14.40 (12.90, 16.90)	14.10 (11.80, 16.28)	0.21
Gamma-glutamyl transferase (GGT, U/L)	13.70 (10.80, 20.75)	12.00 (9.53, 16.53)	**0.001**
Total bilirubin (TBil, μmol/L)	10.00 (8.00, 13.10)	9.50 (7.23, 12.40)	0.553
Direct bilirubin (DBil, μmol/L)	4.20 (3.30, 5.08)	4.05 (3.03, 5.08)	0.617
Indirect bilirubin (IBil, μmol/L)	6.20 (4.25, 8.30)	5.25 (3.83, 7.05)	0.346
Total bile acids (TBA)	2.0 (1.1, 3.1)	1.6 (1.1, 3.0)	0.875
Monocyte percentage (%)	6.20 (5.40, 7.28)	6.10 (5.15, 7.10)	0.065
Eosinophil percentage (%)	0.90 (0.58, 1.43)	0.90 (0.43, 1.50)	**0.003**
Basophil percentage (%)	0.40 (0.20, 0.50)	0.30 (0.20, 0.50)	0.842
Lymphocyte count	1.83 (1.54, 2.12)	1.70 (1.34, 2.04)	0.335
Eosinophil count	0.07 (0.04, 0.10)	0.07 (0.03, 0.11)	**0.014**
Basophil count	0.03 (0.02, 0.04)	0.02 (0.02, 0.04)	0.783
Hemoglobin (Hb)	129 (125, 136)	127.5 (121.25, 134)	0.141
Mean corpuscular volume (MCV, fL)	88.25 (86.15, 90.13)	88.25 (86.30, 90.40)	0.818

Bold values indicate statistical significance (*P* < 0.05). Data are presented as mean ± standard deviation (SD) for normally distributed continuous variables, and median [interquartile range, IQR] for non-normally distributed continuous variables. Comparisons were performed using independent-samples t-test or Mann–Whitney *U-*test as appropriate. A *P* value < 0.05 was considered statistically significant.

Multivariable binary logistic regression analysis was conducted with missed miscarriage coded as the event of interest (Table 2). After adjustment for age, BMI, systolic blood pressure, ALT, GGT, and eosinophil count, SERPINA3 remained independently associated with missed miscarriage (adjusted OR = 1.018, 95% CI: 1.010–1.025, *P* < 0.001). In addition, systolic blood pressure was inversely associated with missed miscarriage (adjusted OR = 0.930, 95% CI: 0.879–0.985, *P* = 0.014), whereas ALT was positively associated with missed miscarriage (adjusted OR = 1.061, 95% CI: 1.009–1.117, *P* = 0.021).

**Table 2:** Multivariable binary logistic regression analysis of factors associated with missed miscarriage.

Variable	B	SE	Wald	df	*P* value	OR [Exp(B)]	95% CI for OR
Age	0.063	0.046	1.876	1	0.171	1.065	0.973–1.166
BMI	0.028	0.094	0.089	1	0.766	1.028	0.855–1.237
Systolic blood pressure	−0.072	0.029	6.075	1	0.014	0.930	0.879–0.985
ALT (U/L)	0.060	0.026	5.321	1	0.021	1.061	1.009–1.117
GGT (U/L)	0.002	0.039	0.002	1	0.968	1.002	0.928–1.082
Eosinophil count	0.671	3.434	0.038	1	0.845	1.956	0.002–1,637.914
SERPINA3 concentration	0.018	0.004	22.060	1	<0.001	1.018	1.010–1.025
Constant	−1.203	3.419	0.124	1	0.725	0.300	—

Presented as mean ± standard deviation (SD) for normally distributed continuous variables, and median [interquartile range, IQR] for non-normally distributed continuous variables. Comparisons were performed using independent-samples *t*-test or Mann–Whitney *U*-test as appropriate. A *P* value < 0.05 was considered statistically significant.

### 4.2 Comparison of SERPINA3 levels among the three groups

**Figure 1:**
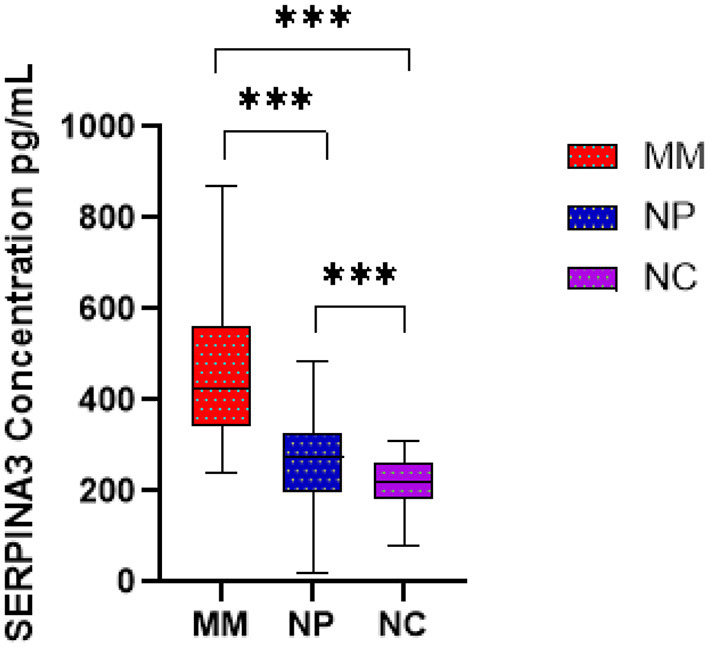
Comparison of plasma SERPINA3 levels between patients with missed miscarriage and the healthy control group. (MM, missed miscarriage group; NP, normal pregnancy group; NC, non-pregnant control group). ^***^indicates *P* < 0.001

As the data were not normally distributed, differences in plasma SERPINA3 levels among the three groups were assessed using the Kruskal–Wallis test. A statistically significant difference was observed among the missed miscarriage group, the normal pregnancy group, and the non-pregnant control group (H = 127.577, df = 2, *P* < 0.001) (Figure 1). *Post-hoc* pairwise comparisons with Bonferroni correction showed significant differences between each pair of groups (all adjusted *P* < 0.05). Plasma SERPINA3 levels were highest in the missed miscarriage group, intermediate in the normal pregnancy group, and lowest in the non-pregnant control group.

### 4.3 Experimental validation of SERPINA3

Age was first compared between the missed miscarriage group and the normal control group. As age did not follow a normal distribution, the Mann-Whitney *U*-test was applied. The results showed no statistically significant difference in age between the two groups (Mann-Whitney *U* = 2,612, Z = −1.393, *P* = 0.164), indicating that the age distributions were comparable between groups. Using the median SERPINA3 concentration in the missed miscarriage group as the cutoff, participants were divided into a high SERPINA3 group and a low SERPINA3 group. The two groups were comparable with respect to age, body mass index, and most clinical and laboratory parameters, with no significant differences observed. Lymphocyte count was significantly higher in the high SERPINA3 group compared with the low SERPINA3 group (*P* = 0.042). Plasma fibrinogen concentration was significantly elevated in the high SERPINA3 group relative to the low SERPINA3 group (*P* = 0.014). In multivariable logistic regression analysis adjusting for age and body mass index, fibrinogen concentration and lymphocyte count remained independently associated with SERPINA3 levels. Although anxiety and depressive symptoms were explored using self-reported GAD-7 and PHQ-9 scores, no significant differences were observed in the present sample. These findings should be interpreted cautiously given the exploratory nature of these assessments and the limited sample size (Tables 3, 4).

**Table 3:** Correlation analysis between high and low concentration SERPINA3 and laboratory indexes.

	High SERPINA3 level (*n* = 45)	Low SERPINA3 level (*n* =46)	*P*
Age (years)			0.224
BMI (kg/m^2^)	23.5 ± 3.8	23.5 ± 4	0.990
Heart rate, bpm	79 ± 9.8	77 ± 7.8	0.34
Systolic blood pressure (mmHg)	116 ± 9	115 ± 10	0.531
Diastolic blood pressure (mmHg)	75 ± 8	75 ± 7	0.735
Urea (mmol/L)	3.31 ± 0.85	3.31 ± 1.05	0.996
Creatinine (μmol/L)	50.63 ± 9.11	50.37 ± 9.49	0.917
AST/ALT ratio	0.99 ± 0.45	1.02 ± 0.46	0.709
Neutrophil (%)	65.25 ± 6.47	67.50 ± 7.10	0.119
Lymphocyte (%)	26.44 ± 5.44	24.71 ± 5.76	0.147
Neutrophil (×10^9^/L)	4.68 ± 1.24	4.73 ± 1.28	0.854
Lymphocyte (×10^9^/L)	1.86 ± 0.50	1.67 ± 0.38	**0.042**
Hematocrit	0.38 ± 0.03	0.37 ± 0.03	0.296
Platelet count (×10^9^/L)	249.10 ± 67.39	252.87 ± 50.45	0.819
Prothrombin time ratio	1.06 ± 0.06	1.06 ± 0.05	0.922
Prothrombin activity	94.99 ± 5.19	94.71 ± 4.01	0.772
Fibrinogen	3.29 ± 0.56	2.96 ± 0.561	**0.014**
Thrombin time (TT)	15.92 ± 0.65	15.99 ± 0.73	0.658
GAD-7	4.00 [2.00, 5.00]	3.50 [2.00, 10.00]	0.493
PHQ-9	2.00 [1.00, 3.00]	4.50 [1.25, 7.75]	0.0988
Blood glucose	4.97 [4.66, 5.22]	5.17 [4.87, 5.26]	0.49
Uric acid	226.00 [203.50, 265.50]	201.00 [180.00, 234.00]	0.071
ALT (U/L)	14.80 [10.70, 28.00]	15.30 [9.95, 29.00]	0.7855
AST (U/L)	14.40 [13.10, 16.30]	14.65 [11.93, 18.12]	0.7986
GGT (U/L)	16.95 [11.97, 25.27]	14.00 [10.70, 18.02]	0.0614
Total bilirubin,μmol/L	10.90 [7.90, 13.80]	9.65 [7.22, 13.20]	0.2763
Direct bilirubin,μmol/L	4.25 [3.08, 5.10]	3.95 [3.02, 5.17]	0.8659
Indirect bilirubin,μmol/L	6.50 [4.50, 9.70]	5.40 [3.82, 7.83]	0.2112
Total bile acids	1.80 [1.25, 2.95]	1.60 [1.10, 2.70]	0.6763
WBC (×10^9^/L)	6.72 [5.94, 8.33]	6.68 [6.12, 7.77]	0.6083
Monocyte (%)	6.15 [5.40, 7.62]	5.65 [4.70, 6.65]	0.079
Eosinophil (%)	1.05 [0.70, 1.73]	1.20 [0.62, 1.82]	0.8367
Basophil (%)	0.35 [0.20, 0.50]	0.35 [0.20, 0.50]	0.9187
Eosinophil (×10^9^/L)	0.08 [0.05, 0.13]	0.08 [0.04, 0.13]	0.7099
Basophil (×10^9^/L)	0.03 [0.02, 0.04]	0.02 [0.01, 0.04]	0.9146
Red blood cell count (RBC) (×10^12^/L)	4.26 [4.04, 4.57]	4.16 [4.07, 4.41]	0.47
Hemoglobin, g/L	127.50 [122.50, 135.25]	126.00 [121.25, 133.00]	0.3303
Mean corpuscular volume (MCV)	87.75 [86.00, 89.55]	88.40 [86.80, 90.42]	0.2635
Preoperative HCG	50,279.97 [16,450.03, 98,490.25]	38,492.43 [20,741.78, 65,167.40]	0.3664
Postoperative HCG	5,846.73 [1,074.67, 9,047.56]	3,700.19 [546.38, 7,636.87]	0.3403
Prothrombin time (PT)	12.60 [12.20, 13.00]	12.60 [12.40, 13.10]	0.725
International normalized ratio (INR)	1.05 [1.02, 1.09]	1.05 [1.04, 1.10]	0.737
Activated partial thromboplastin time (APTT)	27.80 [26.40, 29.30]	27.15 [26.12, 29.08]	0.9172

Data are presented as mean ± standard deviation (SD) for normally distributed continuous variables, and median [interquartile range, IQR] for non-normally distributed continuous variables. Comparisons were performed using independent-samples *t*-test or Mann–Whitney *U*-test as appropriate. *P* < 0.05 was considered statistically significant.

**Table 4:** Multivariable logistic regression analysis of factors associated with SERPINA3 subgroup status within the missed miscarriage group.

Variable	B	SE	Wald	df	*P* value	OR [Exp(B)]	95% CI for OR
Fibrinogen concentration	−1.934	0.693	7.786	1	**0.005**	0.145	0.037–0.562
Lymphocyte count	−1.818	0.781	5.423	1	**0.020**	0.162	0.035–0.750
BMI	0.165	0.085	3.758	1	0.053	1.179	0.998–1.393
Age	−0.029	0.056	0.281	1	0.596	0.971	0.871–1.083
Constant	6.157	3.488	3.115	1	0.078	471.865	—

Presented as mean ± standard deviation (SD) for normally distributed continuous variables, and median [interquartile range, IQR] for non-normally distributed continuous variables. Comparisons were performed using independent-samples *t*-test or Mann–Whitney *U-*test as appropriate. A *P* value < 0.05 was considered statistically significant.

### 4.4 Correlation and linear regression analysis of SERPINA3 levels

SERPINA3 concentration was positively correlated with GGT (*r* = 0.341, *P* = 0.001), WBC (*r* = 0.223, *P* = 0.034), eosinophil percentage (*r* = 0.221, *P* = 0.037), lymphocyte count (*r* = 0.312, *P* = 0.003), and eosinophil count (*r* = 0.307, *P* = 0.003). Furthermore, linear regression analysis revealed that GGT and eosinophil count were independently associated with SERPINA3 concentration.

### 4.5 Diagnostic performance of SERPINA3 based on ROC analysis

**Figure 2:**
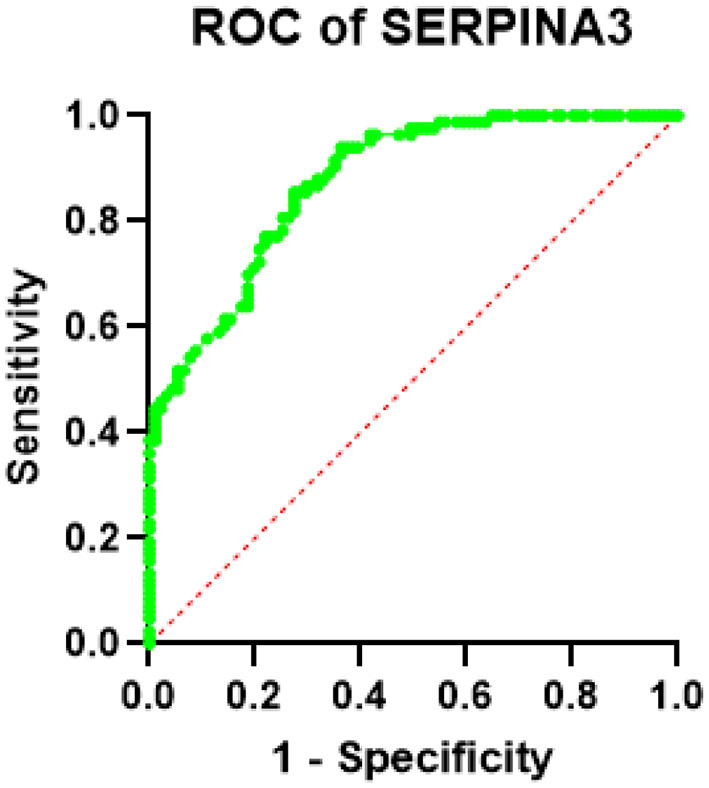
Receiver operating characteristic (ROC) curve of plasma SERPINA3 for discriminating missed miscarriage from normal pregnancy. The area under the curve (AUC) was 0.8751 (*P* < 0.0001), with a sensitivity of 85.54% and a specificity of 72.53%.

ROC analysis indicated that plasma SERPINA3 showed good discriminatory ability between patients with missed miscarriage and normal pregnancy, with an AUC of 0.8751 (95% CI: 0.7641 to 0.9153, *P* < 0.0001), sensitivity of 85.54%, and specificity of 72.53% (Figure 2).

## 5 Discussion

This study demonstrates that circulating SERPINA3 levels are markedly elevated in women with missed miscarriage during early pregnancy, exceeding those observed in both the normal pregnancy group and non-pregnant controls. A stepwise distribution across the three groups suggests that pregnancy itself may be accompanied by upregulation of SERPINA3, with a further increase after the occurrence of missed miscarriage. SERPINA3 showed strong discriminative performance for missed miscarriage versus controls (AUC = 0.8751). Within the missed miscarriage group, higher SERPINA3 levels were accompanied by increased lymphocyte counts and fibrinogen concentrations. Taken together, these findings support SERPINA3 as a promising candidate circulating biomarker and suggest that missed miscarriage may involve systemic inflammation as well as metabolic/oxidative stress–related alterations.

SERPINA3 is a prototypical acute-phase reactant, primarily synthesized in the liver and inducible by pro-inflammatory cytokines. By inhibiting neutrophil- and mast cell–associated proteases, SERPINA3 may influence leukocyte activation, extracellular matrix turnover, and vascular remodeling—processes that are integral to implantation and placentation (25, 31). Persistent inflammation or dysregulated immune control in early pregnancy may disrupt the decidual microenvironment and increase the risk of pregnancy failure (32–34). Therefore, the elevation of SERPINA3 observed in our cohort suggests that abnormal inflammatory activation may already be present even in the absence of overt clinical symptoms such as vaginal bleeding or abdominal pain.

Gamma-glutamyl transferase (GGT) participates in glutathione metabolism and is commonly regarded as an indirect indicator of oxidative stress and hepatobiliary metabolic status (35). In the present study, ALT and GGT levels were lower in the normal pregnancy group than in the missed miscarriage group, whereas the AST/ALT ratio was higher. Moreover, SERPINA3 was positively correlated with GGT, and this association remained independent in multivariable analyses. These results imply that SERPINA3 may reflect not only inflammatory activation but also oxidative stress and metabolic dysregulation.

Hematological findings further supported an inflammatory phenotype. Both the eosinophil percentage and absolute eosinophil count differed between missed miscarriage and normal pregnancy, and SERPINA3 correlated positively with eosinophils, total white blood cell count, and lymphocyte count. In multiple linear regression analyses, eosinophil count remained independently associated with SERPINA3 levels. Although eosinophils are traditionally linked to allergic responses, they can also contribute to inflammatory amplification and cytokine-mediated tissue responses (36).

With respect to coagulation, we did not observe significant differences in routine coagulation indices between early-pregnancy missed miscarriage and normal pregnancy, consistent with clinical experience that overt coagulopathy typically arises after prolonged retention of pregnancy tissue. However, within the missed miscarriage group, higher SERPINA3 levels were also associated with higher fibrinogen concentrations, and this relationship remained significant after adjustment for age and BMI. Given that fibrinogen is also an acute-phase reactant and pregnancy is inherently a hypercoagulable state, this association is more likely to reflect concomitant activation of inflammatory and coagulation pathways. Longitudinal monitoring will be important to clarify their temporal relationship.

Blood pressure is susceptible to contextual and physiological variability, and evidence regarding the association between early-pregnancy blood pressure and miscarriage risk remains inconsistent (37, 38). Likewise, the AST/ALT ratio, ALT, and GGT are influenced by pregnancy-related physiological changes, metabolic status, and oxidative stress. Accordingly, these findings are better interpreted as accompanying changes rather than direct drivers of SERPINA3 elevation.

We also assessed participants' psychological conditions via the GAD-7 and PHQ-9 scales. No notable disparities in anxiety or depression scores were observed between the high and low SERPINA3 groups, suggesting that anxiety and depression did not interfere with SERPINA3 levels in our research. This aligns with the cross-sectional nature of our study, as emotional distress in patients tends to arise following diagnosis, rather than existing prior to missed miscarriage.

Several limitations should be acknowledged. First, chromosomal testing of products of conception was not performed; thus, stratified analyses by fetal karyotype were not possible, and residual confounding related to genetic factors cannot be fully excluded. As embryonic chromosomal abnormalities account for approximately 56%−60% of missed miscarriages (13), the pathophysiology of aneuploidic miscarriage is fundamentally distinct from euploidic miscarriage linked to maternal immune or inflammatory disturbances. Elevated SERPINA3 in the present study may represent a secondary acute-phase response to retained necrotic pregnancy tissue, irrespective of fetal karyotype, rather than a primary pathogenic driver of euploidic pregnancy failure. This important distinction constrains interpretation of its mechanistic role. Second, the sample size was relatively small and derived from a single center. Third, SERPINA3 was measured at a single time point, and no tissue-level assessments were conducted. Finally, unmeasured infectious, immunologic, and environmental factors may also have affected systemic inflammatory indices.

Furthermore, the cross-sectional design of this study entails that blood samples were collected at the time of missed miscarriage diagnosis. Consequently, we cannot determine whether elevated SERPINA3 precedes and predicts subsequent pregnancy loss, or instead arises as a downstream consequence of inflammatory necrosis induced by prolonged retention of the non-viable embryo. This temporal uncertainty means that the predictive capacity of SERPINA3 for impending missed miscarriage remains unvalidated.

## 6 Conclusion

In conclusion, plasma SERPINA3 levels are significantly elevated in first-trimester missed miscarriage and display a graded increase across non-pregnant controls, normal pregnancy, and missed miscarriage. SERPINA3 demonstrates strong discriminatory capacity between missed miscarriage and controls and is independently associated with inflammatory and oxidative stress–related parameters, including GGT, eosinophil count, lymphocyte count, and fibrinogen. These findings suggest that SERPINA3 reflects a systemic inflammatory–metabolic response accompanying early pregnancy failure.

## Data Availability

The original contributions presented in the study are included in the article/supplementary material, further inquiries can be directed to the corresponding authors.
